# Optimal asymmetry and other motion parameters that characterise high-quality female dance

**DOI:** 10.1038/srep42435

**Published:** 2017-02-09

**Authors:** Kristofor McCarty, Hannah Darwin, Piers L. Cornelissen, Tamsin K. Saxton, Martin J. Tovée, Nick Caplan, Nick Neave

**Affiliations:** 1 Department of Psychology, Faculty of Health & Life Sciences, Northumbria University, Newcastle upon Tyne, NE1 8ST, UK; 2 School of Psychology, College of Social Science, University of Lincoln, Lincolnshire, LN6 7TS, UK; 3 Department of Sport, Exercise & Rehabilitation, Faculty of Health & Life Sciences, Northumbria University, Newcastle upon Tyne, NE1 8ST, UK

## Abstract

Dance is a universal human behaviour that is observed particularly in courtship contexts, and that provides information that could be useful to potential partners. Here, we use a data-driven approach to pinpoint the movements that discriminate female dance quality. Using 3D motion-capture we recorded women whilst they danced to a basic rhythm. Video clips of 39 resultant avatars were rated for dance quality, and those ratings were compared to quantitative measurements of the movement patterns using multi-level models. Three types of movement contributed independently to high-quality female dance: greater hip swing, more asymmetric movements of the thighs, and intermediate levels of asymmetric movements of the arms. Hip swing is a trait that identifies female movement, and the ability to move limbs asymmetrically (i.e. independently of the other) may attest to well-developed motor control, so long as this limb independence does not verge into uncontrolled pathological movement. We also found that the same level of dance quality could be predicted by different combinations of dance features. Our work opens avenues to exploring the functional significance, informational content, and temporal sequencing of the different types of movement in dance.

Dance has been found in every human culture studied, serves no immediate survival function, and is frequently expressed in courtship contexts^1^,^2^,^3^. Accordingly, dance is of interest to researchers working on the evolutionary significance of human behaviour because it appears to function, at least in part, as a human courtship display that can serve to attract potential partners^3^. Traits that can attract potential partners have been the subject of extensive research. As such, we now know much about what is attractive in various physical traits including facial appearance, body shape, and vocal qualities, and about how attractive qualities tend to point us towards partners who can provide direct and indirect reproductive benefits^4^,^5^,^6^. The form and significance of attractive dance, however, has been less well studied, and this limits our understanding of its role in human courtship and partner selection.

Research has shown that judges are able to evaluate qualities that might indicate certain aspects of potential partner quality merely from dance movements. For example, socially desirable personality traits, including extraversion, conscientiousness, and emotional stability, have been found to correspond positively to women’s ratings of men’s dance attractiveness^7^,^8^. Traits related to risk-taking are attractive in male partners^9^, and also seem to predict more attractive dance movements^10^. Further, men with stronger handgrip (a proxy for upper body strength), another attractive male feature^11^, are rated as using more attractive dance movements^12^,^13^,^14^. In women, there is some evidence that dance attractiveness might reflect fertility: women’s dance is rated more attractive during high-fertility than low-fertility^15^, and female lap-dancers earn more tips around ovulation^16^.

In order to fully understand what dance movements can convey to potential partners, we need to know which dance movements are appealing. Here, we focus on women’s dance movements. Women dance to entertain men in many cultures^1^. A study in a nightclub found that female dancers were more likely to attract male attention than vice versa^2^, and men pay greater visual attention to attractive than unattractive dancers^17^. Men’s and women’s dance moves are different: gender can be identified from dance movement patterns at fairly high rates by both adult and child raters^18^. Men’s and women’s dance moves also have different significations: only in men, and not in women, does handgrip strength predict dance attractiveness^12^. Although we know something of the types of movement that are attractive in men’s dances^13^,^19^, no functionally-motivated studies have analysed the patterns of movements that improve positive perceptions of female dance.

Here, we use a data driven approach to understand the form of high-quality female dance, with the objective of opening avenues for the understanding of the functional significance and informational content of dance movements. We used motion-capture technology to record the movements of young women as they danced to a drum beat. We rendered their movement patterns onto computer avatars, thereby retaining their distinguishing movements, but removing all information about their individual appearance. We obtained ratings from other men and women of 39 of these avatars in terms of their dance quality, and compared those ratings to quantified measurements of the dancers’ movements.

## Results

In this study we set α = 0.05 and all statistical tests were two-tailed.

### Comparison of male and female ratings

The split-half reliabilities for the mean dance quality rating per image were r = 0.60, p < 0.0001 for both male and female raters. The mean male and female ratings per image were significantly correlated (r = 0.77, p < 0.0001). Together, these results suggest that both sexes were in good agreement about the rank ordering of dance quality.

### Rhythmicity of dance

**Figure 1:**
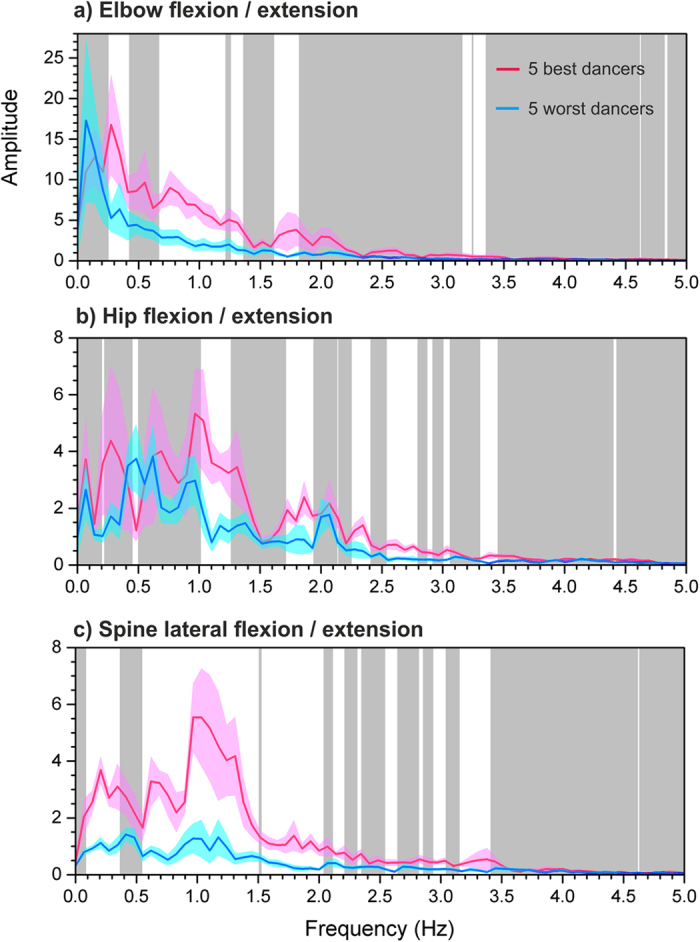
Fast Fourier transform graphs to show the averaged amplitude of joint oscillation as a function of frequency for (**a**) the elbows, (**b**) the hips and (**c**) the spine. The 5 highest-rated dancers are represented by the pink line and the 5 lowest-rated dancers are represented by the blue line. The pink and blue shaded regions in each graph represent the mean amplitudes at each frequency +/−1 standard error of the mean. White background regions indicate that the vertical separation (i.e. the difference in oscillatory amplitude at that frequency) between the two groups of dancers is statistically significant at p < 0.05 corrected for multiple comparisons.

Dance is characterised by oscillatory movement that is linked to the beat of the music. These beat-linked movements should be apparent within frequency-domain descriptions of the time-domain recordings as peaks at discrete frequency bands. We predicted that the movements of better dancers would be tied more closely to the beat of the music, and thus would give rise to peaks of greater magnitude. To test this, we compared the frequency-domain descriptions of the five best and five worst rated dancers. We focussed on the recordings of the elbow, hip and spine joint angles, because these were identified as the salient biomechanical variables that discriminated between the two groups of dancers (see Methods). We used Origin Pro 2016 to compute fast Fourier transforms (FFTs) from the time series of these joint angles. To standardize this comparison, a fixed window of 14.5 sec was selected from each of the 39 dance sequences. A low pass Hanning filter was applied to the time series with the upper frequency set at the Nyquist limit for these data of 100 Hz. Figure 1 plots the average FFT amplitude for the elbow (a), hip (b) and spine (c) as a function of oscillatory frequency, separately for the highest-rated and lowest-rated groups of dancers.

The dance music heard by the dancers was played at a rate of 125 beats per minute, which corresponds to a frequency of 2.08 Hz. To illustrate, if one imagines 4 drum beats to the bar at 125 bmp, the hip swing from the left to the right and back again would correspond to a frequency of ~1 Hz. Figure 1 demonstrates that the highest-rated dancers showed significantly higher peaks of oscillatory activity, particularly for the spine and hips, at around a frequency of ~1 Hz and less so at ~0.5 Hz and 0.25 Hz. This is what we would expect if the movements of the highest rated dancers were better synchronised to the beat of the music than those of the lowest rated dancers.

### Using focal movement parameters to explain differences in rated dance attractiveness

We used PROC MIXED (SAS v9.4) to fit five separate multi-level models to explain dance quality on the basis of five movement parameters: asymmetric arm movements, (i.e. the right arm moving independently of the left), asymmetric thigh movements (i.e. the right upper leg moving independently of the left), hip swing, amount of arm movement, and amount of thigh movement (see Methods for more information on quantification of these parameters). Multilevel models have the advantage of modelling variability in both raters and stimuli (dancers) simultaneously as fully crossed random effects. Traditional analyses that model variability in stimuli or raters alone are known to inflate Type I error (see e.g. refs 20 and 21).

As explanatory variables in each model, we initially included: (i) trial order, (ii) the sex of the rater (Sex), (iii) the movement parameter (standard deviation: SD), (iv) second order polynomial terms for the movement parameters (SD^2^) where appropriate, (v) the interaction between sex and movement parameters (Sex × SD). The distributions for hip swing and thigh movement failed the Shapiro-Wilks test of normality (W = 0.84, p < 0.001 and W = 0.92, p = 0.008 respectively), and therefore these data were logarithmically transformed for all analyses. In addition, based on significant reductions in -2log likelihood, we modelled intercept variation for both raters and dancers by specifying an ‘unstructured’ variance-covariance structure for each in the model’s G-matrix. For all five models initially, there were no statistically significant effects of trial order, and so this variable was excluded from the final analysis. Detailed model outputs are reported in Table 1. The dummy coding in the model for sex used males as the reference.

**Figure 2:**
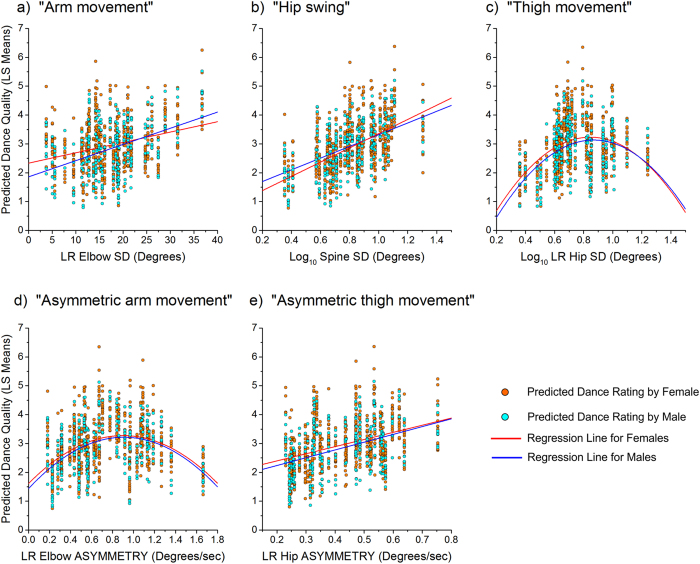
Plots of the LSmean dance quality, estimated from each of the five final models, plotted as a function of five focal movement parameters. Orange and cyan dots show LSmean estimates of dance quality for each female and male rater, respectively. The red and blue lines represent the regression lines (linear or polynomial) for female and male raters, respectively.

Both male and female raters judged that more attractive dances contained greater arm movement and hip swing, and more asymmetric thigh movements (Table 1 and Fig. 2). Higher quality dances also contained intermediate quantities of two of our focal parameters: thigh movement, and asymmetric arm movements (see polynomial relationships in Table 1 and Fig. 2). That is, dances that contained some thigh movement, and some asymmetric arm movements, were rated more attractive than dances that contained high or low quantities of these movements. In addition, we found a significant main effect of rater sex, together with a significant interaction between rater sex and arm movement, in relation to dance quality ratings. This combination of effects indicates that the influence of arm movement on dance quality ratings was stronger for female than male raters for low values, equivalent at intermediate values, and stronger for male than female raters at high values.

### Contribution of focal movement parameters to dance quality ratings

**Figure 3:**
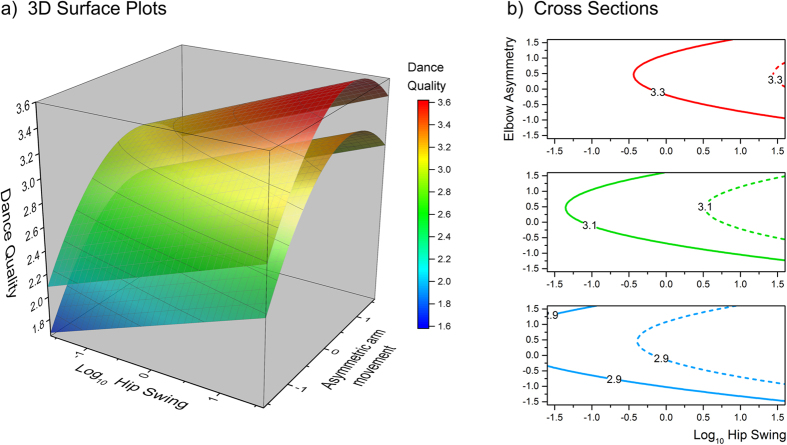
(**a**) 3D surface plots to show the effects of hip swing (x-axis) and asymmetric arm movements (y-axis) on dance quality ratings (z-axis), at a z-score of +1 SD for asymmetric thigh movement (top surface plot) and a z-score of −1 SD for asymmetric thigh movement (bottom surface plot). (**b**) shows cross-sections through these surfaces to illustrate how three dance quality ratings (2.9 in blue, 3.1 in green and 3.3 in red) can be achieved via different combinations of hip swing and asymmetric arm movements, at +1 SD of asymmetric thigh movement (solid lines) and at −1 SD of asymmetric thigh movement (dashed lines).

The foregoing analyses characterize the relationship between dance quality ratings and the five dance movement metrics, when computed independently from separate models. A critical question, however, is which of these metrics optimally explain dance quality ratings when competing against each other *simultaneously* in the same analysis. We therefore used PROC MIXED (SAS v9.4) to fit a final mixed model which initially contained rater gender together with all five dance movement metrics as explanatory variables. We optimized the final model by finding a solution which: (i) minimized −2 log-likelihood, and (ii) only retained explanatory variables that were statistically significant at p < =0.05. In order to allow meaningful comparison of the magnitudes of model regression weights, we also centred all continuous explanatory variables by converting them to z-scores. The model outcome is reported in Table 2, which shows that linear terms for both asymmetric thigh movements and hip swing, as well as polynomial terms for asymmetric arm movements, were together sufficient to explain dance quality ratings according to our optimization criteria. That is, dances were rated more highly if they contained more hip swing, more asymmetric thigh movements, and moderately asymmetric arm movements. This model is illustrated in Fig. 3.

Figure 3 illustrates how the same level of dance quality is predicted by different combinations of the three movement metrics. For example, the middle cross-section in Fig. 3b shows that when asymmetric thigh movement is +1, a dance quality rating of 3.1 can be achieved at the highest hip swing values together with the lowest asymmetric arm movement (i.e. the solid green curve). At mid-range values for asymmetric arm movement, less hip swing is required to achieve the same result. However, at the highest values for asymmetric arm movement, there is a need for greater hip swing again. When asymmetric thigh movement is set to the lower level of −1, the regime to achieve a dance quality rating of 3.1 undergoes a rightward shift such that greater hip swing is required for all values of asymmetric arm movement (i.e. the dashed green curve).

## Discussion

Rather than positing *a priori* assumptions about the (potentially infinite) movement types that would be highly rated, we used a data-driven approach to uncover attractive movement types and forms. Three types of movement made independent contributions to female dance quality: higher ratings were awarded to (1) greater hip swing (i.e. to a greater standard deviation of the spine lateral flexion/extension angle); (2) more asymmetric thigh movements (i.e. to a greater standard deviation of the difference between the differentiated left hip joint angle and the differentiated right hip joint angle time series); and (3) moderate asymmetric arm movements (calculated in the same way as the thigh movements, but with reference to the elbow joint in place of the hip joint). The optimum quantity of asymmetric arm movement was around the median value of our sample (Fig. 2).

The movements that distinguish high-quality dancers might have functional significance. Female and male walkers differ in terms of hip sway^22^, and viewers use hips alongside shoulders to determine gender^23^. Accordingly, hip swing and asymmetric thigh movement might be an emphatically feminine trait. Hand movement may also distinguish male and female dancers, with women tending to move their hands more than men^24^. Fast and changeable movement of the hands has been linked to positive affect in dancers^25^. Typically feminine traits tend to enhance rated attractiveness, and may help viewers identify fecund females^4^,^5^,^6^; fertility (indexed by a woman’s phase in the ovulatory cycle) might be apparent in movement^15^,^16^.

Asymmetric movements of the arms and thighs might also have biological significance. It is more challenging to undertake different movements than the same movement in two limbs simultaneously, and so asymmetric movements attest to high-quality motor control^26^. In our sample, we found that the degree of movement was important; the highest rated dances demonstrated intermediate rather than low or high levels of asymmetric arm movement. Both of these options might indicate poor motor control. Indeed, in extreme cases, hypokinesia can indicate serious medical conditions such as Parkinson’s disease, just as hyperkinesia can indicate Huntington’s disease or Tourette’s syndrome. Thus to be attractive, the quantity of movement has to fall within a specific window. The significance of and tension between symmetric and asymmetric movement patterns is something that has received a great deal of attention in the literature on the aesthetics of dance^27^.

We also found that when making dance quality judgements, there are several movement configurations that result in the same dance rating, consistent with cue trading^28^. For example, the graph in Fig. 3b shows that when making a minimum amount of asymmetric arm movement (~0.5), a dancer could in principal choose between: (i) low levels of hip swing (~−1.25) combined with high asymmetric thigh movement (~+1.0) or (ii) high levels of hip swing (~0.5) combined with low asymmetric thigh movement (~−1.0) to achieve the same dance quality rating of 3.1. This suggests that rather than there being a single optimal configuration of movements leading to a given dance rating, there may be multiple routes to achieving the same outcome by trading off one movement attribute for another. Given that dance constitutes a voluntary movement pattern, there is also the question not only of whether a particular dancer would tend to use the same set of ‘sub-routines’ whenever they dance, but also whether a given observer would assign the same relative weightings to different movement components each time they rate dance. In general, evidence of cue trading fits with other findings in human mate choice. For example, women have been shown to trade off creative intelligence against wealth when choosing potential partners^29^.

Previous research has shown that male dance quality can be predicted by variability in the amplitude of neck and trunk movements together with the speed of movement of the right knee^19^. Here, using cutting-edge motion-capture technology combined with powerful multi-level models, we have uncovered a set of specific movement parameters associated with perceived female dance quality. We are now in a position to further consider the potential signalling value of such movement patterns, for example by assessing the links between the movements and female reproductive quality (health, fertility etc).

While male and female raters were generally consistent in their ratings, we found some statistically significant sex differences (Fig. 2). Heterosexual male raters might be evaluating the dancers in terms of their potential as partners, while heterosexual female raters might be assessing the dancers in terms of their potential as competitors.

Future work might examine whether dance attractiveness differs according to variables that distinguish individuals in terms of their value or potential as a partner or competitor. These variables could include those that have previously been shown to reliably predict perceived attractiveness between individuals, such as symmetry and femininity^4^,^5^,^6^. They could also include variables that change within individuals and also affect attractiveness ratings and reproductive potential, such as health, age, and ovulatory cycle phase^30^,^31^,^32^. Previous work has found that the physical traits of a single individual tend to be awarded similar ratings of attractiveness, suggesting that traits function as a single ornament of partner quality^33^,^34^. Dance might be another part of this ornament.

A limitation to the current study is that all the dance metrics we identified were extracted from a standard 14.5 second window and collapsed across time. Consequently, we were only in a position to investigate how variable combinations in the relative magnitudes of these summary metrics for arm, spine and leg movement predicted dance quality. In future work, it could be informative to obtain longer dance sequences and apply moving time windows to these sequences in order to investigate how dynamic variation of these, and other metrics, over time may make additional contributions to explaining dance quality. Nevertheless, our work opens avenues to exploring the functional significance, informational content, and temporal dynamics of the different types of movement apparent in dance.

## Methods

The study protocol was approved by the Faculty of Health & Life Sciences Ethics Committee, in accordance with Northumbria University Research and Governance Guidelines. All participants provided informed consent to participation.

### Stimuli

Our dancers consisted of heterosexual females aged 18–30 (mean = 21.55, s.d. = 3.29). All were students at a university in the north-east of England. Our initial sample numbered 40 women, but due to technical issues, we obtained dance ratings for only 39 of them. No participant was a professional dancer, nor experiencing any injuries or health problems that would affect her movements. To record movements, 39 14-mm reflective markers were attached at key anatomical features and joints specified by the Vicon Plug-In-Gait marker set. Elbow, ankle, and wrist widths, and leg lengths, were also measured, so that the actual body movements could be extrapolated from the recorded positions of the reflective markers. Dance movements were captured with a 12-camera optical motion capture system (Vicon MX, Vicon, Oxford) running Nexus v1.7 software calibrated to measure marker position within 2mm at a frequency of 200 Hz and a resolution of 1600 × 1280 pixels. After an initial calibration ‘T-pose’, participants were asked to dance for 30 sec to a basic 125 bpm drum rhythm. They were given no instructions on how they should dance.

A kinematic model (Plug-In-Gait, Vicon, Oxford) was used to generate three dimensional joint angles for each dancer’s knee, hips, trunk (spine), elbows, neck, shoulders and wrists. Vicon Nexus 1.7 was used to extract the data for each dancer to a.txt file in raw format for further analysis. The motion capture data from each dancer were used to animate a virtual character (avatar) using Autodesk MotionBuilder 2011 (Autodesk Inc., San Rafael, CA, USA). This process removed any features that distinguished the dancers (e.g. appearance, height, body size), allowing us to isolate the effects of the dance movements alone on ratings of dance quality (see supplementary videos of a good [S1] and bad dancer [S2] online).

### Dance ratings

Raters were recruited through a variety of means including links on social media and advertisements within a university psychology department, and this recruitment process is perhaps reflected in the gender bias of the raters. Raters were invited to take part if they were aged 18 or over and were heterosexual. Ratings were carried out in an online study hosted on Qualtrics (www.qualtrics.com). Fifty-seven males (mean age = 30.88, SD = 13.68) and 143 females (mean age = 26.54, SD = 10.49) provided dance ratings based on a 15 sec section of each of the 39 dance avatars shown in an 800 × 600 pixel window. Raters viewed a random subset of 5 avatars, and rated the quality of each dance on a 1-7 Likert-type scale (1 = extremely bad dancer to 7 = extremely good dancer). The drum rhythm that the dancers heard was not played to the raters. Each avatar was rated a minimum of 20 times (mean = 32.0, s.d. = 3.85).

### Identification of movement quality

Our analysis strategy was to perform a first-pass expert assessment to identify the movements that discriminated the highest and lowest rated dances, and then to use a multi-level modelling analysis to uncover whether those parameters predicted ratings of the dance quality of our complete set of dance stimuli.

### Selection of focal movement parameters

**Figure 4:**
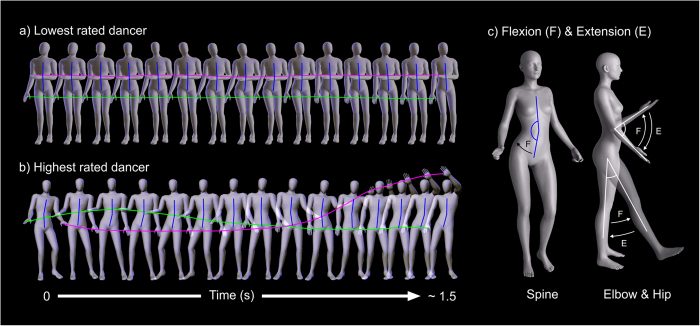
A schematic of the dance movements of (**a**) the lowest rated dancer and (**b**) the highest rated dancer over 1.5 sec. Panel (**c**) illustrates anatomical flexion/extension of the spine, elbow and hip respectively. The green and magenta lines in (**a**,**b**) track the movements of the right and left hands, respectively, over time; these movements are indexed by the degree of elbow flexion/extension. The vertical blue line illustrates the degree of spine lateral flexion/extension. As can be seen in the top sequence, the lowest rated dancer shows little arm movement, and minimal spine flexion/extension. In contrast, the highest rated dancer shows far greater movement of both arms and spine, and the two are arms are moving independently, not together.

First, we wanted to identify which joint movements discriminated the dances perceived to be of highest and lowest quality. To do this, we calculated the mean rating awarded to each dancer, selected the five highest and lowest rated dancers, and subjected those two groups to visual inspection by two expert observers, trained in the biomechanical analysis of human movement. These two observers were blind to the aims of the experiment and made their judgements independently from each other. Both observers were fully aware that the Plug-in-Gait kinematic model renders 22 joint angles. We asked them to identify which, if any, of those joint angles best distinguished between the dance movements of the five best- and five worst- rated dancers. Both observers were in perfect agreement that hip flexion/extension, elbow flexion/extension and spine lateral flexion/extension were the salient biomechanical variables (illustrated in Fig. 4).

As can be seen in Fig. 4c, spine flexion/extension represents the extent to which the upper body does or does not create a straight line with the lower body, as the hips and pelvis pendulate to the side. Elbow flexion/extension captures the degree to which the lower arm moves with respect to the upper arm. Hip flexion/extension captures the degree to which the upper leg moves away from and towards the vertical line created by the trunk. Figure 1a,b also illustrate that independence of movement patterns between the left and right side appeared to discriminate the best and worst dances: that is, the extent to which movement was asymmetric between left and right arm, or between left and right leg.

### Quantification of focal movement parameters: creation of the variables for analysis

First, we identified the longest continuous time window common to all forty dance sequences that contained complete motion capture data. This standard window lasted 14.5 seconds and was used for all subsequent analyses. To quantify both the overall extent of movement of the three joint angles (elbow, hip and spine) for each dancer, as well as the degree of asymmetry in their arm and leg movements, we calculated two metrics from the joint angle time series. The first was the standard deviation (SD) of the distributions of joint angle samples, calculated separately for the 3 joint angles from each dancer, over the course of the standard window. At the 200 Hz sampling rate of the Vicon system, this gave us ~2900 joint angle samples per limb. Our notion was that the more a dancer moved their elbows / hips / spine, the higher should be the SD of the respective joint angle. A similar analysis strategy has been described previously as a way to estimate the variability of binocular vergence angles for binocularly recorded eye movements during reading^35^. Since the Pearson correlations between the SD metric for left and right hips and left and right elbows were r = 0.79, p < 0.0001 and r = 0.72, p < 0.0001 respectively, we averaged these values together for further analysis. This process provided three values for each dancer: SD of spine lateral flexion/extension over time (referred to henceforth as “hip swing”), SD of elbow joint movement over time (“arm movement”), and SD of hip joint movement over time (“thigh movement”).

The second metric captured dynamic changes in asymmetry between the left and right elbow movements, and the left and right hip movements. To do this, we computed the standard deviation (SD) of the difference between the *differentiated* time series for the left joint angle (elbow or hip) and right joint angle (elbow or hip). Higher values therefore represented greater independence of movement between left and right limbs. We found that if we used the undifferentiated signal (i.e. where the time series corresponds to joint angle as a function of time, rather than joint angular velocity with time) we could obtain apparently high asymmetry values in some dancers, even though both arms/hips moved very little. For example, if a dancer simply held one arm in a higher fixed position than the other we could obtain such false positive results, and this was avoided by computing the SD of the difference in the differentiated (velocity) traces. This process gave rise to two values for each dancer, referred to above as “asymmetric arm movements” and “asymmetric thigh movements”.

## Additional Information

**How to cite this article:** McCarty, K. *et al*. Optimal asymmetry and other motion parameters that characterise high-quality female dance. *Sci. Rep.* **7**, 42435; doi: 10.1038/srep42435 (2017).

**Publisher's note:** Springer Nature remains neutral with regard to jurisdictional claims in published maps and institutional affiliations.

## Supplementary Material

Supplementary Video 1

Supplementary Video 2

## Figures and Tables

**Table 1:** Output from five independent multi-level models testing the influence of dance metrics on dance quality.

Model Parameter	F-value (DF)	Z-value	p-value	Estimate	95% CI	−2Log likelihood
Empty model						2840.9
Log _10_ Thigh movement						
Sex	0.13 (1, 828)		0.26	0.13	−0.57–0.82	2756.7
SD	10.03 (1, 37.4)		0.0031	11.23	4.06–18.41	
SD^2^	8.72 (1, 37.5)		0.0054	−6.59	−11.10−2.07	
Sex × SD	0.04 (1, 712)		0.85	−0.084	−0.94–0.78	
Observer covariance		6.43	<0.0001	0.49		
Dancer covariance		3.82	<0.0001	0.38		
Asymmetric thigh movement						
Sex	0.41 (1, 815)		0.41	0.19	−0.39–0.77	2757.4
SD	10.18 (1, 39.4)		0.0028	2.77	0.93–4.61	
Sex × SD	0.23 (1, 732)		0.23	−0.30	−1.52–0.92	
Observer covariance		6.41	<0.0001	0.49		
Dancer covariance		3.81	<0.0001	0.40		
Arm movement						
Sex	4.54 (1, 751)		0.033	0.52	0.041–1.0052	2822.8
SD	9.40 (1, 39.8)		0.0039	0.061	0.026–0.096	
Sex × SD	5.06 (1, 747)		0.025	−0.026	−0.049−0.0033	
Observer covariance		6.54	<0.0001	0.50		
Dancer covariance		3.88	<0.0001	0.41		
Asymmetric arm movements						
Sex	4.81 (1, 623)		0.029	0.46	0.029–0.05	2815.8
SD	14.58 (1, 39.1)		0.0005	4.84	2.41–7.26	
SD^2^	11.67 (1, 39.2)		0.0015	−2.44	−3.88–−0.99	
Sex × SD	6.36 (1, 738)		0.012	−0.56	−1.00–−0.12	
Observer covariance		6.55	<0.0001	0.49		
Dancer covariance		3.82	<0.0001	0.35		
Log_10_ Hip swing						
Sex	0.18 (1, 849)		0.67	0.15	−0.52–0.82	2815.6
SD	24.82 (1, 40.5)		<0.0001	2.32	1.28–3.36	
Sex × SD	0.07 (1, 747)		0.78	−0.11	−0.87–0.66	
Observer covariance		6.50	<0.0001	0.49		
Dancer covariance		3.68	<0.0001	0.28		

**Table 2:** Output from optimized multi-level model, initially including all five dance movement metrics and gender of the rater as explanatory variables.

Model Parameter	F-value (DF)	Z-value	p-value	Estimate	95% CI	−2Log likelihood
Empty model						2840.9
Asymmetric thigh movement	3.94 (1, 39.8)		0.054	0.13	−0.0024–0.26	2814.7
Log_10_ hip swing	6.40 (1, 40.3)		0.015	0.17	0.034–0.31	
Asymmetric arm movement	4.39 (1, 39.3)		0.042	0.14	0.0049–0.27	
Asymmetric arm movement^2^	5.39 (1, 38.4)		0.025	−0.13	−0.24–−0.016	
Observer covariance		6.75	<0.0001	0.25		
Dancer covariance		3.49	0.0002	0.094		
